# Supplementation‐induced increase in circulating omega‐3 serum levels is not associated with a reduction in depressive symptoms: Results from the MooDFOOD depression prevention trial

**DOI:** 10.1002/da.23092

**Published:** 2020-08-26

**Authors:** Carisha S. Thesing, Yuri Milaneschi, Mariska Bot, Ingeborg A. Brouwer, Matt Owens, Ulrich Hegerl, Margalida Gili, Miquel Roca, Elisabeth Kohls, Ed Watkins, Marjolein Visser, Brenda W. J. H. Penninx

**Affiliations:** ^1^ Department of Psychiatry, Amsterdam Public Health Research Institute de Boelelaan, Amsterdam UMC Vrije Universiteit Amsterdam Amsterdam The Netherlands; ^2^ Department of Health Sciences, Faculty of Science and the Amsterdam Public Health research institute Vrije Universiteit Amsterdam Amsterdam the Netherlands; ^3^ Department of Psychology University of Exeter Exeter United Kingdom; ^4^ Department of Psychiatry, Psychosomatics, and Psychotherapy Goethe‐Universität Frankfurt Frankfurt a.M. Germany; ^5^ Institut Universitari d'Investigació en Ciències de la Salut, Idisba, Rediapp University of Balearic Islands Palma de Mallorca Spain; ^6^ Department of Psychiatry and Psychotherapy, Medical Faculty University of Leipzig Leipzig Germany

**Keywords:** MooDFOOD, omega‐3, prevention, subclinical depression, supplement

## Abstract

**Background:**

There is ambiguity on how omega‐3 (n‐3) polyunsaturated fatty acids (PUFAs) are associated with depression, and what the temporality of the association might be. The present study aimed to examine whether (intervention‐induced changes in) n‐3 PUFA levels were associated with (changes in) depressive symptoms.

**Methods:**

Baseline, 6‐ and 12‐month follow‐up data on 682 overweight and subclinically depressed persons from four European countries that participated in the MooDFOOD depression prevention randomized controlled trial were used. Participants were allocated to four intervention groups: (a) placebos, (b) placebos and food‐related behavioral activation therapy (F‐BA), (c) multinutrient supplements (fish oil and multivitamin), and (d) multinutrient supplements and F‐BA. Depressive symptoms were measured using the inventory of depressive symptomatology. PUFA levels (µmol/L) were measured using gas chromatography. Analyses were adjusted for sociodemographics, lifestyle, and somatic health.

**Results:**

Increases in n‐3 PUFA, docosahexaenoic acid, and eicosapentaenoic acid levels over time were significantly larger in the supplement groups than in placebo groups. Change in PUFA levels was not significantly associated with the change in depressive symptoms (*β* = .002, SE = 0.003, *p* = .39; *β* = .003, SE = 0.005, *p* = .64; *β* = .005, SE = 0.005, *p* = .29; *β* = −.0002, SE = 0.0004, *p* = .69). Baseline PUFA levels did not modify the intervention effects on depressive symptoms.

**Conclusions:**

In overweight and subclinical depressed persons, multinutrient supplements led to significant increases in n‐3 PUFA levels over time, which were not associated with changes in depressive symptoms. Multinutrient supplements do not seem to be an effective preventive strategy in lowering depressive symptoms over time in these at‐risk groups.

## 1 INTRODUCTION

Omega‐3 (n‐3) polyunsaturated fatty acids (PUFAs) consist of, for example, α‐linolenic acid (ALA), eicosapentaenoic acid (EPA) and docosahexaenoic acid (DHA) and can be mainly found in fatty fish, some other seafood, and some nuts and seeds (James, Gibson, & Cleland, 2000; Simopoulos, 1999). ALA is a so‐called essential fatty acid as humans must ingest ALA through their diet (e.g., leafy green vegetables and in flaxseed and canola oils), or through supplements as our bodies seem to require ALA for good health but cannot synthesize ALA by itself (Young & Conquer, 2005). Omega‐6 (n‐6) PUFAs consist of, for example, linoleic acid and arachidonic acid, which are, for example, found in plant and vegetable seeds and oils as found in margarine and many processed foods (James et al., 2000; Simopoulos, 1999).

Some observational studies have shown that low n‐3 PUFA plasma levels are more often found in patients with depressive disorders than in healthy individuals, but other studies do not confirm this (Lin, Huang, & Su, 2010; Smith, Beilin, & Mori, 2011; Wani, Bhat, & Ara, 2015). Several potential biochemical mechanisms could explain the association between PUFAs and depressive disorders (Smith et al., 2011). For a long time, it was thought that the anti‐inflammatory property of n‐3 PUFAs may mitigate the overactive immune system associated with several disorders (Young & Conquer, 2005), whereas n‐6 PUFAs were generally seen as proinflammatory (Husted & Bouzinova, 2016) because n‐3 and n‐6 PUFAs compete for the same substrates. However, it has recently been discovered that the biochemistry of n‐3 and n‐6 PUFA is much more nuanced, with both n‐3 and n‐6 PUFAs having proinflammatory and anti‐inflammatory properties, and neither is “all good” or “all bad” (Nasir & Bloch, 2019). In addition to inflammatory activities that could underlie depressive and anxiety disorders, a decrease in dietary DHA is related to a decrease in cortical serotonin and dopamine (Young & Conquer, 2005), and these neurotransmitters have been implicated in the etiology of depressive disorders (Smith et al., 2011; Young & Conquer, 2005). In addition, fatty acids are implicated as regulators of gene transcription within the central nervous system (Alessandri et al., 2004; Smith et al., 2011) and may play a role in neural membrane fluidity and receptor binding (Owen, Rees, & Parker, 2008; Smith et al., 2011; Stahl, Begg, Weisinger, & Sinclair, 2008). For instance, DHA can affect neurological function by modulating neurotransmission, neurogenesis, myelination, and more (Weiser, Butt, & Mohajeri, 2016). As not all studies have found this association between low n‐3 PUFA levels and depressive disorders, a possible role of potential third factors (i.e., residual confounding factors) has been suggested, such as biological stress (Thesing, Bot, Milaneschi, Giltay, & Penninx, 2018b) or psychological vulnerabilities (Thesing et al., 2018a), although these could also be considered mediators.

Despite not all studies finding a cross‐sectional association between n‐3 PUFAs and depressive disorders, systematic reviews, and meta‐analysis of the randomized controlled trials (RCTs) that investigated n‐3 PUFA supplementation for the treatment of depressive disorders have concluded that overall a beneficial effect can be found (Appleton, Rogers, & Ness, 2010; Appleton, Sallis, Perry, Ness, & Churchill, 2015; Bloch & Hannestad, 2012; Grosso et al., 2014; Mocking et al., 2016). This seems especially true for EPA and in patients taking antidepressants, as shown by a recent meta‐analysis involving 13 studies and 1,233 participants (Mocking et al., 2016). However, large heterogeneity has been found in effect sizes, which could possibly be explained by methodological differences between the studies (e.g., sample backgrounds and supplement types, doses, and duration). Hence, the clinical significance of the found effect is still questionable. Some meta‐analytic evidence (Appleton et al., 2010; Bloch & Hannestad, 2012) suggests a potential role of clinical depression characteristics in the efficacy of n‐3 PUFA supplementation; studies including more‐severely depressed patients tend to show higher efficacy of n‐3 PUFA supplementation to treat depression than studies including less‐severe‐depressed patients.

It is important to make a distinction between treatment studies and prevention studies, as the factors involved in the development of a disorder in at‐risk individuals may be different than those factors involved in the course of a depressive disorder in patients. Recently, we showed in the multicountry MooDFOOD depression prevention trial among 1,025 overweight adults with subclinical depressive symptoms that multinutrient supplementation (including n‐3 PUFA fish oil supplements and a multivitamin) and a food‐related behavioral activation therapy both did not reduce the onset of episodes of major depressive disorder during 1 year (Bot et al., 2019). As the supplements contained n‐3 PUFAs and the food‐related behavioral activation therapy promoted eating fish at least three times a week, it was hypothesized that both interventions would lead to an increase in n‐3 PUFA serum levels in participants, which was hypothesized to lead to a reduction in depressive symptoms and a decreased risk for the onset of a new depressive disorder episode. However, against expectations, the supplements resulted in slightly poorer depressive and anxiety symptoms scores compared with the placebo.

To date, n‐3 and n‐6 PUFA serum levels are finally available for a large part of the MooDFOOD sample which provides us with the opportunity to examine whether these interventions resulted in a significant change in n‐3 and n‐6 PUFA levels and whether these changes are related to a subsequent change in depressive symptoms over time. Furthermore, it could also be that the interventions were only effective in participants with low baseline n‐3 PUFA levels and high baseline n‐6 PUFA levels, as these subjects may benefit most from the interventions, also in terms of the impact on depressive symptoms. Consequently, this study examined whether both supplementation, as well as food‐related behavioral activation, lead to an increase in serum PUFA levels and whether such change is associated with a change in depressive symptoms. We also aimed to examine whether participants with lower n‐3 PUFA levels or higher n‐6 PUFA levels at baseline were most responding to the interventions (e.g., in terms of depression symptoms over time).

## 2 METHODS

### 2.1 Study design and sample

The MooDFOOD depression prevention trial was designed to measure the feasibility and effectiveness of two different nutritional strategies to prevent a new episode of major depressive disorder (MDD) in high‐risk overweight persons with subclinical symptoms of depression. This study was a 2 × 2 factorial RCT performed between July 30, 2015, and October 13, 2017, in four European countries (Germany, Spain, United Kingdom, and the Netherlands). For full details of trial design and protocol, see Roca et al. (2016) and for trial results, see Bot et al. (2019). Ethics approval was provided by the human research ethics boards of the study sites. All participants provided written informed consent. A total of 1,025 participants were recruited by the four study sites (located in Leipzig, Germany; Palma de Mallorca, Spain; Exeter, United Kingdom; and Amsterdam, the Netherlands). Main eligibility criteria were: age between 18 and 75 years, body mass index (BMI; calculated as weight in kilograms divided by height in meters squared) between 25 and 40 kg/m^2^, and having at least mild depressive symptoms as operationalized by Patient Health Questionnaire scores of 5 or higher (Kroenke, Spitzer, & Williams, 2001) but having no current MDD episode in the past 6 months (Mini International Neuropsychiatric Interview 5.0; Sheehan et al., 1998). All eligible participants were invited to visit one of the study sites for a baseline interview. The interview, physical measurements, and blood sampling were conducted by trained research assistants or nurses. Furthermore, participants completed self‐report questionnaires. Follow‐up assessments in which blood was assessed took place at 3, 6, and 12 months. For the analyses of the current study, only participants with available data on at least one PUFA measurement were included (*N* = 682). Included participants were significantly older, were physically more active, drank more glasses of alcohol per week, were more often higher educated, and were less often current smokers than excluded participants (*n* = 343). The investigation was carried out in accordance with the latest version of the Declaration of Helsinki (clinicaltrials.gov; trial registration number: NCT02529423).

### 2.2 Measurements

#### 2.2.1 Fatty acid measurements

Blood samples were collected at all four sites, stored at −80°C and shipped to Reference Laboratory in Barcelona (Spain) for analyses using gas chromatography. For each participant, in total 2 ml of blood serum was used for assessment of n‐3 PUFA, DHA, EPA, and n‐6 PUFA (in µmol/L). Compared to the other countries, a smaller number of participants from Germany provided blood samples due to logistic reasons that were not related to the randomization of the interventions. At baseline, 6 months, and 12 months follow‐up, the number of participants with available blood data was 673, 479, and 477, respectively.

#### 2.2.2 Severity of depression

Severity of depressive symptoms was assessed using the 30‐item self‐report Inventory of Depressive Symptomatology questionnaire (IDS‐SR_30_, range 0–84), with higher scores indicating higher severity (Rush, Gullion, Basco, Jarrett, & Trivedi, 1996). At baseline, 6 months, and 12 months of follow‐up, the number of participants with available IDS‐SR_30_ data was 994, 747, and 757, respectively.

#### 2.2.3 Intervention groups

Patients received either multinutrient supplements (1,412 mg of EPA and DHA; ratio, 3:1), 30 μg of selenium, 400 μg of folic acid, and 20 μg of vitamin D3 coupled with 100 mg of calcium) provided in two pills per day taken daily for 1 year, or matching placebo pills. The food‐related behavioral activation therapy (F‐BA) consisted of a protocol‐based intervention that incorporated standard behavioral activation approaches. Behavioral activation is effective in depression treatment (Ekers et al., 2014) and includes self‐monitoring, functional analysis, and activity schedule. Food‐related behavioral activation applied these proven techniques to improve mood by changing dietary habits, food‐related behaviors (e.g., snacking), increasing positive behaviors, and emphasizing a Mediterranean‐style diet, which has been related to reduced depression onset (Molendijk, Molero, Ortuño Sánchez‐Pedreño, Van der Does, & Angel Martínez‐González, 2018). A maximum of 21 sessions was provided (15 individual sessions and 6 in a group) for 1 year. No active (e.g., attention) control condition was provided in those receiving no therapy. In total 1,025 participants were randomized over four intervention groups taking into account site and history of depressive disorders: (a) placebo without F‐BA (*n* = 257), (b) placebo with F‐BA (*n* = 256), (c) supplement without F‐BA (*n* = 256), and (d) supplement with F‐BA (*n* = 256).

#### 2.2.4 Covariates

Sociodemographic covariates were age, gender, education, and site (The Netherlands, Germany, Spain and England). Education was divided into low (no education, primary education, or lower secondary education), middle (upper secondary education; postsecondary nontertiary education, short‐cycle tertiary education), and high (bachelor's degree or higher or equivalent level). Somatic health and lifestyle covariates were BMI (weight in kg/length in m^2^), physical activity, smoking (never, former, current), alcohol use, diabetes mellitus (yes/no), heart disease (yes/no), number of other chronic somatic disorders (continuous). As we expected that diagnosis of diabetes (Mahendran et al., 2013) and heart disease (Würtz et al., 2015) would have stronger associations with PUFA's, we separated these from other chronic somatic diseases. Physical activity was measured with the following question from the Short Questionnaire to Assess Health: “How many days a week do you exercise (combination of bicycling, gardening, do sport or household work) for at least 30 min?” and expressed in days/week (Wendel‐Vos, Schuit, Saris, & Kromhout, 2003).

### 2.3 Statistical analyses

Frequency and descriptive statistics were used to describe the baseline sample.

The first analyses examined the association between the four intervention groups and longitudinal trajectories of n‐3 PUFA, DHA, EPA, and n‐6 PUFA, for which linear mixed models with a random intercept at the subject level were used, taking into account the correlation across repeated measures of the same subject (Twisk et al., 2018). As compared to traditional repeated measures analyses, mixed‐effects models have the advantage of making use of the data of all the subjects despite missing observations. The second analysis examined whether, over time, change in n‐3 PUFA, DHA, EPA, and n‐6 PUFA levels was associated with the change in depression severity. As suggested by Twisk (2013), we used modeling of change scores for both PUFA levels and depression severity (6‐month follow‐up values minus baseline values, and 12‐month follow‐up values minus 6‐month follow‐up values) to create variables reflecting within‐subject change over the two blocks of time in a long data set. This data set was analyzed using generalized estimating equation analyses as reported in Twisk (2013), in which 0‐ to 6‐month change scores in PUFA levels were used as predictors for 0‐ to 6‐month change scores in depressive symptoms, and 6‐ to 12‐month change scores in PUFA levels were used as predictors for 6‐ to 12‐month change scores in depressive symptoms. Finally, the third analysis estimated the association between the treatment group (supplements yes/no; F‐BA yes/no) and depressive symptoms over time, while examining effect modification by baseline PUFA levels by adding treatment group‐by‐PUFA baseline levels interaction terms in linear mixed models already including the related main terms. Analyses were conducted using IBM SPSS statistics software, version 25 (IBM Corp., Armonk, NY). Statistical significance threshold was set at *α* < .05, two‐tailed.

## 3 RESULTS

### 3.1 Sample description

Descriptive statistics of the study sample are presented in Table 1. The mean age of all participants included at baseline (*N* = 682) was 48.6 years (standard deviation = 12.4) and 75.5% were female. Table S1 shows all Spearman's correlation coefficients between all PUFA measures at baseline, 6 months, and 12 months of follow‐up.

**Table 1:** Descriptive statistics of the study sample (*n* = 682)

	Placebo without F‐BA (*n* = 164)	Placebo with F‐BA (*n* = 175)	Supplement without F‐BA (*n* = 174)	Supplement with F‐BA (*n* = 169)
Demographic variables				
Age, mean (*SD*)	48.0 (12.1)	48.5 (12.0)	49.2 (13.2)	48.6 (12.2)
Female (%)	72.0	74.9	78.7	76.3
Education (%)				
Low	10.4	9.7	14.9	10.7
Middle	42.7	48.0	42.0	40.8
High	47.0	42.3	43.1	48.5
Site of data collection (%)				
Germany	6.1	5.7	8.6	8.9
United Kingdom	38.4	34.3	35.6	34.3
Spain	20.7	24.6	21.8	22.5
Netherlands	34.8	35.4	33.9	34.3
Somatic health and lifestyle				
BMI, median (p25–75)	30.9 (27.6–33.6)	30.7 (27.8–33.0)	30.8 (28.0–33.0)	30.8 (28.1–33.7)
Diabetes, yes (%)	4.3	4.0	4.6	4.1
Heart disease, yes (%)	4.9	4.6	8.0	6.5
No. of other chronic somatic disorders^a^, mean (*SD*)	0.16 (0.53)	0.22 (0.56)	0.26 (0.73)	0.30 (0.86)
Smoking (%)				
Never	42.8	44.0	48.3	43.8
Former	41.5	40.6	38.5	37.9
Current	15.9	15.4	13.2	18.3
Alcohol use (glasses/week), median (p25–75)	1.04 (0.19–3.78)	2.42 (0.19–8.23)	1.04 (0.19–8.23)	1.04 (0.31–3.77)
Physical activity (SQUASH), median (p25–75)	4.00 (2.00–6.00)	4.00 (1.00–5.00)	4.00 (2.00–6.00)	4.00 (2.00–6.75)
Fatty acids				
N‐3 PUFA (µmol/L)				
Baseline, median (p25–75)	226 (147–283)	209 (131–320)	220 (138–298)	198 (136–298)
6‐month follow‐up, median (p25–75)	210 (158–312)	249 (157–345)	296 (199–410)	290 (186–400)
12‐month follow‐up, median (p25–75)	221 (170–307)	250 (185–317)	297 (211–379)	274 (182–366)
DHA (µmol/L)				
Baseline, median (p25–75)	141 (96.0–181)	138 (92.5–204)	145 (92.0–192)	127 (86.0–194)
6‐month follow‐up, median (p25–75)	140 (103–197)	155 (105–220)	163 (114–210)	156 (105–214)
12‐month follow‐up, median (p25–75)	146 (110–198)	160 (118–215)	170 (129–206)	155 (110–207)
EPA (µmol/L)				
Baseline, median (p25–75)	75.0 (44.0–111)	73.0 (34.0–112)	77.0 (45.0–109)	74 (42.5–106)
6‐month follow‐up, median (p25–75)	75.0 (52.0–117)	86.0 (54.0–124)	137 (74.0–186)	117 (71.0–192)
12‐month follow‐up, median (p25–75)	77.0 (48.5–109)	81.0 (59.0–112)	122 (78.0–185)	109 (65.5–176)
N‐6 PUFA (µmol/L)				
Baseline, median (p25–75)	2,824 (2,427–3,340)	2,793 (2,385–3,265)	2,807 (2,496–3,249)	2,808 (2,386–3,393)
6‐month follow‐up, median (p25–75)	2,724 (2,311–3,379)	2,875 (2,534–3,366)	2,714 (2,337–3,327)	2,722 (2,300–3,199)
12‐month follow‐up, median (p25–75)	2,854 (2,507–3,506)	2,997 (2,576–3,485)	2,763 (2,474–3,141)	2,868 (2,435–3,248)
Depression characteristics				
IDS‐SR‐30				
Baseline, median (p25–75)	20.0 (14.0–26.0)	21.0 (13.0–29.5)	21.0 (15.0–30.0)	21.0 (15.0–28.0)
6‐month follow‐up, median (p25–75)	12.0 (8.00–20.0)	12.0 (7.00–19.0)	14.0 (8.00–23.0)	15.5 (9.00–21.0)
12‐month follow‐up, median (p25–75)	14.0 (7.00–21.0)	11.0 (6.00–17.5)	12.0 (8.00–21.5)	12.0 (7.00–19.0)
History of MDD, yes (%)	36.0	36.6	39.7	38.5

Abbreviations: AD, antidepressant; BMI, body mass index; DHA, docosahexaenoic acid; EPA, eicosapentaenoic acid; F‐BA, food‐related behavioral activation therapy; IDS‐SR‐30, Inventory of depressive symptomatology self‐report questionnaire with 30 questions; MDD, major depressive disorder; N‐3, omega‐3; N‐6, omega‐6; PUFA, polyunsaturated fatty acids; p, percentile; *SD*, standard deviation; SQUASH, Short Questionnaire to Assess Health.

a Other than diabetes or heart disease.

### 3.2 Group differences in PUFA levels over time

**Figure 1:**
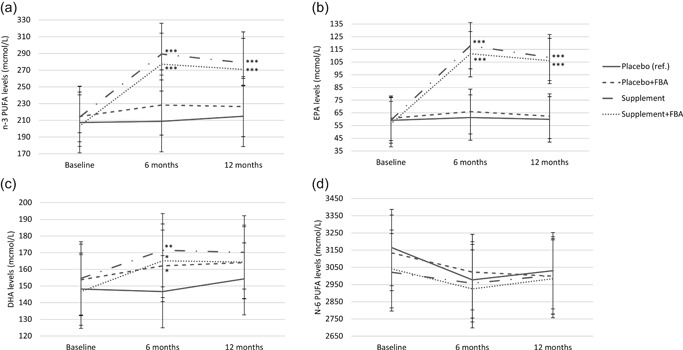
N‐3 PUFA, DHA, EPA, and n‐6 PUFA levels at baseline, 6‐, and 12‐month follow‐up in the group with the placebos and no F‐BA (*n* = 164), the group with placebo and F‐BA (*n* = 175), the group with the supplements without F‐BA (*n* = 174), and the group with the supplements and with F‐BA (*n* = 169; total sample at baseline *N* = 682). DHA, docosahexaenoic acid; EPA, eicosapentaenoic acid; F‐BA, food‐related behavioral activation therapy; N‐3, omega‐3; N‐6, omega‐6; PUFA, polyunsaturated fatty acids

Figure 1 depicts the estimated mean n‐3 PUFA, DHA, EPA, and n‐6 PUFA levels for all four intervention groups derived from a linear mixed model (Table S2) adjusted for all sociodemographic, lifestyle, and somatic health covariates. At the 6‐month follow‐up, the supplement group without F‐BA, and the supplement group with F‐BA, had higher n‐3 PUFA (Cohen's *d* = 0.27, *p* < .001; Cohen's *d* = 0.23, *p* < .001), EPA (Cohen's *d* = 0.38, *p* < .001; Cohen's *d* = 0.34, *p* < .001), and DHA (Cohen's *d* = 0.14, *p* = .001; Cohen's *d* = 0.10, *p* = .013) levels compared to the placebo without F‐BA group. At the 12‐month follow‐up, the supplement group without F‐BA and the supplement group with F‐BA, still had higher levels of n‐3 PUFA (Cohen's *d* = 0.21, *p* < .001, Cohen's *d* = 0.19, *p* < .001) and EPA levels (Cohen's *d* = 0.33, *p* < .001; Cohen's *d* = 0.31, *p* < .001) as compared to the placebo without F‐BA group, but only the supplement group without F‐BA maintained significantly higher DHA level (*d* = .09, *p* = .032). The placebo group with F‐BA had significantly higher levels of DHA (*d* = 0.09, *p* = .033) only at a 6‐month follow‐up, as compared to the placebo group without F‐BA. No significant differences in mean n‐6 PUFA levels across the four groups were found at any time point.

### 3.3 Longitudinal associations between PUFAs and depression severity

As shown in Table 2, after adjustment for all sociodemographic, lifestyle, and somatic health covariates, the changes in n‐3 PUFA, DHA, EPA, and n‐6 PUFA levels over time were not significantly associated with changes in depressive symptoms severity (*β* = .002, SE = 0.003, *p* = .39; *β* = .003, SE = 0.005, *p* = .64; *β* = .005, SE = 0.005, *p* = .29; *β* = −.0002, SE = 0.0004, *p* = .69). These nonsignificant associations were consistent between baseline and 6 months of follow‐up and between 6 and 12 months of follow‐up for all PUFA measures (PUFA‐by‐time interaction terms: n‐3 PUFA *p* = .69, DHA *p* = .89, EPA *p* = .53, and n‐6 PUFA *p* = .81, respectively).

**Table 2:** Longitudinal associations between change in n‐3 PUFA, DHA, EPA, and n‐6 PUFA levels and change in depression severity (*n* = 682)

	Change in depression severity		
	*β*	SE	*p* Value
Change in n‐3 PUFA	.002	0.003	.39
Time (months)			
0–6 (ref.)			
6–12	5.27	0.625	<.001
Interaction effects			
N‐3 PUFA by 0–6 months (ref.)			
N‐3 PUFA by 6–12 months	.002	0.005	.69
Change in DHA	.003	0.005	.64
Time(months)			
0–6 (ref.)			
6–12	5.19	0.603	<.001
Interaction effects			
DHA by 0–6 months (ref.)			
DHA by 6–12 months	.001	0.010	.89
Change in EPA	.005	0.005	.29
Time (months)			
0–6 (ref.)			
6–12	5.34	0.638	<.001
Interaction effects			
EPA by 0–6 months (ref.)			
EPA by 6–12 months	.005	0.007	.53
Change n‐6 PUFA	−.0002	0.0004	.69
Time (months)			
0–6 (ref.)			
6–12	5.20	0.599	<.001
Interaction effects			
N‐6 PUFA by 0–6 months (ref.)			
N‐6 PUFA by 6–12 months	−.0002	0.001	.81

*Note*: Analyses are adjusted for age, gender, site, education, body mass index, smoking, alcohol use, physical activity, diabetes mellitus, heart disease, and other chronic somatic disorders.

Abbreviations: DHA, docosahexaenoic acid; EPA, eicosapentaenoic acid; n‐3, omega‐3; n‐6, omega‐6; PUFA, polyunsaturated fatty acids.

### 3.4 Modification of intervention effects on depressive symptoms by baseline PUFA levels

Table 3 reports the results of mixed models adjusted for all sociodemographic, lifestyle, and somatic health covariates, examining whether the effect of multinutrient and n‐3 PUFA fish oil supplements on depressive symptoms over time is modified by baseline n‐3 PUFA, DHA, EPA, and n‐6 PUFA levels. Subjects taking supplements, as compared to those on placebo, tended to have higher although statistically not significant, depressive symptoms over the entire follow‐up (*β* = 1.13, SE = 0.621, *p* = .070; *β* = 1.12, SE = 0.621, *p* = .070; *β* = 1.13, SE = 0.620, *p* = .068; *β* = 1.13, SE = 0.622, *p* = .070, respectively). Baseline n‐3 PUFA, DHA, EPA, and n‐6 PUFA did not modify the effect of the supplements (*p* = .40, *p* = .29, p = .67, and *p* = .84, respectively). This was consistent over time (e.g,. all baseline PUFA‐by‐intervention‐by‐time interaction terms, *p* > .05). Subjects in the groups with F‐BA had depressive symptoms severity comparable to subjects in the groups without F‐BA, and the effect of the F‐BA on depressive symptoms over time was also not modified by baseline n‐3 PUFA, DHA, EPA, and n‐6 PUFA levels (results not shown).

**Table 3:** Longitudinal associations between supplement use and baseline n‐3 PUFA, DHA, EPA or n‐6 PUFA levels with depression severity (*n* = 682)

	Depression severity over time		
	*β*	SE	*p* Value
Baseline n‐3 PUFA	.004	0.003	.25
Group			
Placebo's (ref.)			
Supplement	1.13	0.621	.07
Interactions			
n‐3 PUFA‐by‐supplement use	−.004	0.005	.40
n‐3 PUFA‐by‐supplement use‐by‐6 months of follow‐up	.002	0.006	.73
n‐3 PUFA‐by‐supplement use‐by‐12 months of follow‐up	.006	0.006	.33
Baseline DHA	.005	0.005	.36
Group			
Placebo's (ref.)			
Supplement	1.12	0.621	.070
Interactions			
DHA‐by‐supplement use	−.009	0.008	.29
DHA ‐by‐supplement use‐by‐6 months of follow‐up	.003	0.010	.78
DHA ‐by‐supplement use‐by‐12 months of follow‐up	.008	0.010	.44
Baseline EPA	.010	0.007	.199
Group			
Placebo's (ref.)			
Supplement	1.13	0.620	.068
Interactions			
EPA‐by‐supplement use	−.005	0.011	.67
EPA ‐by‐supplement use‐by‐6 months of follow‐up	.005	0.014	.69
EPA ‐by‐supplement use‐by‐12 months of follow‐up	.016	0.013	.24
Baseline n‐6 PUFA	.0001	0.001	.89
Group			
Placebo's (ref.)			
Supplement	1.13	0.622	.070
Interactions			
n‐6 PUFA‐by‐supplement use	.0002	0.001	.84
n‐6 PUFA‐by‐supplement use‐by‐6 months of follow‐up	.001	0.001	.24
n‐6 PUFA‐by‐supplement use‐by‐12 months of follow‐up	.001	0.001	.27

*Note*: Analyses are adjusted for age, gender, site, education, body mass index, smoking, alcohol use, physical activity, diabetes mellitus, heart disease, and other chronic somatic disorders.

Abbreviations: DHA, docosahexaenoic acid; EPA, eicosapentaenoic acid; n‐3, omega‐3; n‐6, omega‐6; PUFA, polyunsaturated fatty acids.

## 4 DISCUSSION

The present study examined associations of n‐3 PUFA (including DHA and EPA) and n‐6 PUFA levels with depressive symptoms using data on 682 participants from the MooDFOOD depression prevention trial (Bot et al., 2019). We found that n‐3 PUFA and EPA levels can be effectively increased by multinutrient supplements, but these increases were not associated with a significant reduction in depressive symptoms over time. Also, baseline PUFA levels did not modify the effect of the multinutrient supplements or the F‐BA. Therefore, our experimental study does not support the hypothesis that n‐3 PUFA levels have a direct causal effect on depressive symptoms.

More specifically, significantly larger increases in n‐3 PUFA and EPA levels were found in the two supplement groups versus the two placebo groups, which was consistent with our expectations. This difference indicates that the n‐3 PUFA supplements were sufficiently used to give a contrast in blood n‐3 PUFA and EPA levels, as was already shown in the adherence (77%) to supplements (Bot et al., 2019). Interestingly, the effect of the supplements tended to level off at 6 months of follow‐up (see Figure 1), which could mean that adherence to supplement intake decreased at that time point, or that there is a ceiling effect, for example, after reaching a certain level of PUFA you cannot increase PUFA levels any more (Maki, Palacios, Bell, & Toth, 2017; Meyer & de Groot, 2017). However, a biological mechanism explaining the specific n‐3 PUFA level at which a ceiling effect is reached has not been proposed yet. Additionally, it should be noted that it has not yet been established what the healthy range is for PUFA levels. As EPA is the main ingredient of these fish oil supplements (ratio EPA:DHA was 3:1) and as conversion of EPA into DHA is low (Simopoulos, 1999), it was expected that DHA would not increase in a similar magnitude, which was confirmed. Perhaps a higher dosage of DHA would be needed to provide a larger change in DHA blood levels. Earlier study designs differ in terms of duration and administration of supplements and in fatty acids measurements which makes comparing effect sizes difficult. The study by Carney et al. (2009) has also found a statistical difference in postsupplementation omega‐3‐index between the supplement group and placebo group, for which we calculated a Cohen's *d* of 1.96. In the present study, for n‐3 PUFA levels after 12 months of supplementation Cohen's *d*s of 0.27 (supplements without F‐BA group) and 0.19 (supplements with F‐BA group) were found when compared to the placebo without F‐BA group.

Diet is an important source for n‐3 and n‐6 PUFAs (Simopoulos, 1999). Although the F‐BA promoted an n‐3 PUFA rich diet (e.g., three times of fish per week), F‐BA did not lead to changes in PUFA serum levels, as the groups with F‐BA did not have significantly higher n‐3 PUFA, DHA, or EPA levels or lower n‐6 PUFA levels at 6‐ or 12‐month follow‐up when compared to the groups without F‐BA, except for higher DHA levels at 6 months of follow‐up in the group with placebo and F‐BA. The fact that we did not find a consistent increase in all n‐3 PUFA measures and at all time points was unexpected, because a self‐reported increased intake of food products high in n‐3 PUFAs (e.g., fatty fish, nuts, and seeds) in the F‐BA groups was found previously in the same MooDFOOD study sample (Grasso et al., 2019). It seems like the increase of intake of products high in n‐3 PUFAs was only moderate and therefore may not have led to substantial increases in all n‐3 PUFAs.

Furthermore, no significant associations were found between change in n‐3 PUFA, DHA, EPA, and n‐6 PUFA levels with a change in depressive symptoms. This is in line with a recent study that applied Mendelian randomization analyses—inferring a causal relationship between modifiable risk factors and disease using genetic variants as a natural experiment—on data from ~500,000 subjects (Milaneschi et al., 2019). Findings provided no evidence of a causal role of n‐3 PUFA on the risk of MDD onset. The question arises what could be the nature of the association between n‐3 PUFA levels and depressive disorders. One explanation could be that low n‐3 PUFA levels may arise when depressive symptoms arise, for example, as an epiphenomenon. Possibly, common third factors, such as dysregulations in biological stress systems (Thesing et al., 2018a) or psychological vulnerabilities (Thesing et al., 2018a), may lead to both a depressive disorder and n‐3 PUFA alterations. A second explanation could be that the direction of the association is the other way around: depressive symptoms may lead, for example, via unhealthy food intake (e.g., consuming fewer products high in n‐3 PUFAs such as fatty fish), to lower n‐3 PUFA plasma levels. A third explanation could be that alterations in PUFA levels are an adaptive process, protecting the body for future damage by oxidative stress, as some PUFAs are highly oxidizable (Assies et al., 2014) and as increased levels of oxidative stress have been found in patients with a depressive disorder (Black, Bot, Scheffer, Cuijpers, & Penninx, 2015). In all three cases, supplementing persons with n‐3 PUFAs to increase n‐3 PUFA levels would not be effective in preventing depression, and in the third case, supplementation may even be harmful (e.g., increase oxidative stress). It is important to note that in the MooDFOOD prevention trial the supplements resulted in slightly poorer depressive and anxiety symptoms scores compared with placebo (Bot et al., 2019). Harmful side effects might have been the reason for this.

As stated earlier, it is important to make a distinction between treatment studies and prevention studies. The three explanations given above for the possible nature of the association between n‐3 PUFA levels and depressive disorder, are not in line with some meta‐analyses of RCTs that do find a beneficial treatment effect of n‐3 PUFA supplementation in depressed patients, although with small effect sizes and, therefore, questionable clinical significance (Appleton et al., 2010, 2015; Grosso et al., 2014; Mocking et al., 2016). This suggests that n‐3 PUFA supplements may be less effective in individuals at risk for depression than in currently depressed patients. Recently, the International Society for Nutritional Psychiatry Research Practice has formulated guidelines for the use of n‐3 PUFA supplementation in the treatment and prevention of MDD (Guu et al., 2019). In response, we argued that there is indeed some evidence from RCTs for the usefulness of n‐3 PUFA supplementation in the treatment of clinically depressed cases, but there is not enough evidence from RCTs on the usefulness of n‐3 PUFA supplementation in the prevention of depression in at‐risk individuals (Thesing, Lamers, Bot, Penninx, & Milaneschi, 2019). The question arises how it can be that n‐3 PUFA supplementation could be effective in the treatment of clinically depressed patients, but not in the prevention of depression in subclinically depressed patients, and maybe even lead to slightly poorer depressive and anxiety symptoms scores compared with placebo (Bot et al., 2019). One explanation could be that the biological mechanisms and related markers (including a chemical imbalance in the brain, e.g., n‐3 PUFA alterations next to neurotransmitter imbalances) of onset are different from those of disease progression. Additionally, other markers (such as nonbiological markers, i.e. life events or personality) may be more involved in the onset of a depressive disorder, whereas n‐3 PUFAs might be involved more in the progression of a depressive disorder. If this is the case, n‐3 PUFA supplements would be inefficient in preventing the onset of depression, but might be effective in the treatment of a current depression.

At last, PUFA levels at the start of the study did not modify the effect of the supplements or the F‐BA on depressive symptoms over time. It was hypothesized that the interventions would be more effective in participants with baseline n‐3 PUFA levels in a lower range, assuming that the multinutrient interventions would improve these levels, and subsequently impact on depressive symptoms. However, there was no evidence to suggest this. Careful selection of participants that might benefit from n‐3 PUFA supplementation (e.g., based on pretreatment PUFA deficiencies) might therefore not be indicated.

A strength of the current study is the randomized and double‐blind 2 × 2 factorial design and the large sample (*N* = 682) of participants that are overweight/obese and have subclinical depressed symptoms with available data on PUFA levels and depressive symptoms using a well‐validated instrument (IDS‐SR_30_). Due to the preventive nature of this trial, we included participants with elevated depressive symptoms who did not meet the criteria for MDD at baseline. The depression symptoms scores at baseline may have been too low to permit any significant findings, that is, a floor effect. Also, on average, due to “regression to the mean” phenomenon and the naturalistic fluctuation of depressive symptoms over time the overall depressive symptomatology went down in all four intervention groups over time. In line with this, the onset of MDD was limited (only 10% developed MDD in our sample), which was lower than expected and, therefore, the power to analyze this indicator was too small (see Bot et al., 2019 for more details). One limitation is the potential generalizability, as we included only participants that were overweight/obese sample. Baseline blood samples were only available for 682 (66.5%) of all MooDFOOD participants. Furthermore, due to drop‐out, only 70% (*n* = 477) of those with baseline blood samples had 12‐month follow‐up data. However, we used statistical methods that allowed us to use the data from all 682 participants at all time points despite missing observations. Fortunately, included and excluded participants did not differ on the severity of depression. Analyses did not further consider food intake, but at baseline, our intervention groups did not differ (Grasso et al., 2019) on this factor. As there is no universally agreed/golden standard reference range for PUFA levels, and as this also depends on measurement aspects including laboratory‐specific analytical procedures, a reference range for low versus high PUFA levels cannot be given or used in the current study.

To summarize, this study shows that n‐3 PUFA and EPA levels can be effectively increased by fish oil supplements, but that these observed changes in n‐3 PUFA, EPA, DHA, and n‐6 PUFA levels over a 6‐month period were not associated with changes in depressive symptoms in the same period, and that baseline PUFA levels do not modify the effects of fish oil supplements on depressive symptoms. This could be one of the many possible explanations for the lack of preventive effect for depressive disorders found in the MooDFOOD depression prevention trial study. It can be concluded that the increase in n‐3 PUFA, DHA, and EPA circulating levels obtained through the n‐3 PUFA supplementation regime implemented in the MooDFOOD study (Bot et al., 2019) is not associated with a reduction in depressive symptoms in persons that have subclinical depressive symptoms. As several previous RCTs did find a beneficial treatment effect of n‐3 PUFA supplementation (Appleton et al., 2010, 2015; Grosso et al., 2014; Mocking et al., 2016), this may mean that PUFA alterations might be more involved in the progression of a depressive disorder rather than in the development of a depressive disorder.

## CONFLICT OF INTERESTS

Miquel Roca has received (non‐related) research funding from Janssen Research and Lundbeck. Brenda W. J. H. Penninx has received (non‐related) research funding from Jansen Research and Boehringer Ingelheim.

## Supporting information

Supporting information

## Data Availability

The data that support the findings of this study are available from the corresponding author upon reasonable request.
