# Supplementary material for: Supplementation‐induced increase in circulating omega‐3 serum levels is not associated with a reduction in depressive symptoms: Results from the MooDFOOD depression prevention trial
Source: Depress Anxiety. 2020 Aug 26;37(11):1079–88. doi: 10.1002/da.23092 (PMC7693241; doi:10.1002/da.23092)
Supplement: Supplementary file 1 — Supporting information [file DA-37-1079-s001.docx]

**Supplements**

Table S1. Spearman correlations between all PUFA levels at baseline, 6 and 12 months of follow-up (n=682).

|  |  | Baseline | | | | 6 months of follow-up | | | | 12 months of follow-up | | | |
| --- | --- | --- | --- | --- | --- | --- | --- | --- | --- | --- | --- | --- | --- |
|  |  | n-3 PUFA | DHA | EPA | n-6  PUFA | n-3 PUFA | DHA | EPA | n-6  PUFA | n-3 PUFA | DHA | EPA | n-6  PUFA |
| Baseline | n-3 PUFA |  |  |  |  |  |  |  |  |  |  |  |  |
|  | DHA | .97** |  |  |  |  |  |  |  |  |  |  |  |
|  | EPA | .94** | .85** |  |  |  |  |  |  |  |  |  |  |
|  | n-6 PUFA | .56** | .56** | .50** |  |  |  |  |  |  |  |  |  |
| 6 months of follow-up | n-3 PUFA | .76** | .73** | .72** | .38** |  |  |  |  |  |  |  |  |
|  | DHA | .78** | .78** | .71** | .43** | .95** |  |  |  |  |  |  |  |
|  | EPA | .67** | .62** | .67** | .30** | .94** | .80** |  |  |  |  |  |  |
|  | n-6 PUFA | .35** | .35** | .31** | .58** | .46** | .53** | .33** |  |  |  |  |  |
| 12 months of follow-up | n-3 PUFA | .72** | .69** | .69** | .36** | .79** | .75** | .74** | .32** |  |  |  |  |
|  | DHA | .74** | .75** | .66** | .40** | .74** | .78** | .63** | .36** | .93** |  |  |  |
|  | EPA | .61** | .55** | .65** | .30** | .74** | .64** | .76** | .24** | .92** | .73** |  |  |
|  | n-6 PUFA | .33** | .32** | .31** | .59** | .34** | .42** | .22** | .63** | .39** | .41** | .33** |  |
| *Note*. **<.01 n-3: omega-3. n-6: omega-6. PUFA: polyunsaturated fatty acids. DHA: docosahexaenoic acid. EPA: eicosapentaenoic acid. | | | | | | | | | | | | | |

Table S2. Longitudinal associations between group and n-3 PUFA, DHA, EPA and n-6 PUFA trajectories over time (n=682).

|  | N-3 PUFA | | |
| --- | --- | --- | --- |
|  | Beta | SE | p-value |
| Group |  |  |  |
| Placebo (ref.) |  |  |  |
| Placebo + F-BA | 7.26 | 11.2 | .52 |
| Supplements | 6.59 | 11.2 | .56 |
| Supplements + F-BA | -4.09 | 11.3 | .72 |
| Time |  |  |  |
| Main effect at baseline (ref.) |  |  |  |
| Main effect time at 6-months follow-up | 1.28 | 9.16 | .89 |
| Main effect time at 12-months follow-up | 7.48 | 9.13 | .41 |
| Interaction effects |  |  |  |
| Placebo + F-BA by 6-months follow-up | 12.3 | 12.5 | .33 |
| Supplements by 6-months follow-up | 74.0 | 12.8 | <.001 |
| Supplements + F-BA by 6-months follow-up | 72.6 | 12.8 | <.001 |
| Placebo + F-BA by 12-months follow-up | 4.30 | 12.5 | .73 |
| Supplements by 12-months follow-up | 57.4 | 12.8 | <.001 |
| Supplements + F-BA by 12-months follow-up | 60.2 | 12.8 | <.001 |
|  | DHA | | |
| Group |  |  |  |
| Placebo (ref.) |  |  |  |
| Placebo + F-BA | 5.58 | 6.53 | .39 |
| Supplements | 6.44 | 6.55 | .33 |
| Supplements + F-BA | -1.44 | 6.60 | .83 |
| Time |  |  |  |
| Main effect at baseline (ref.) |  |  |  |
| Main effect time at 6-months follow-up | -1.52 | 4.89 | .76 |
| Main effect time at 12-months follow-up | 6.14 | 4.87 | .21 |
| Interaction effects |  |  |  |
| Placebo + F-BA by 6-months follow-up | 9.91 | 6.67 | .143 |
| Supplements by 6-months follow-up | 18.4 | 6.84 | .007 |
| Supplements + F-BA by 6-months follow-up | 20.0 | 6.81 | <.001 |
| Placebo + F-BA by 12-months follow-up | 4.18 | 6.67 | .53 |
| Supplements by 12-months follow-up | 9.45 | 6.81 | .165 |
| Supplements + F-BA by 12-months follow-up | 11.6 | 6.84 | .091 |
|  | EPA | | |
| Group |  |  |  |
| Placebo (ref.) |  |  |  |
| Placebo + F-BA | 1.67 | 5.72 | .77 |
| Supplements | 0.24 | 5.74 | .97 |
| Supplements + F-BA | -2.59 | 5.78 | .65 |
| Time |  |  |  |
| Main effect at baseline (ref.) |  |  |  |
| Main effect time at 6-months follow-up | 2.24 | 5.24 | .67 |
| Main effect time at 12-months follow-up | 0.82 | 5.22 | .88 |
| Interaction effects |  |  |  |
| Placebo + F-BA by 6-months follow-up | 3.03 | 7.16 | .67 |
| Supplements by 6-months follow-up | 56.4 | 7.33 | <.001 |
| Supplements + F-BA by 6-months follow-up | 52.8 | 7.31 | <.001 |
| Placebo + F-BA by 12-months follow-up | 0.80 | 7.16 | .91 |
| Supplements by 12-months follow-up | 48.5 | 7.30 | <.001 |
| Supplements + F-BA by 12-months follow-up | 48.8 | 7.34 | <.001 |
|  | n-6 PUFA | | |
| Group |  |  |  |
| Placebo (ref.) |  |  |  |
| Placebo + F-BA | -31.6 | 68.7 | .65 |
| Supplements | -26.6 | 69.0 | .70 |
| Supplements + F-BA | -46.4 | 69.5 | .50 |
| Time |  |  |  |
| Main effect at baseline (ref.) |  |  |  |
| Main effect time at 6-months follow-up | -53.2 | 57.0 | .35 |
| Main effect time at 12-months follow-up | 134.6 | 56.8 | .028 |
| Interaction effects |  |  |  |
| Placebo + F-BA by 6-months follow-up | 76.8 | 78.0 | .32 |
| Supplements by 6-months follow-up | 7.42 | 79.9 | .93 |
| Supplements + F-BA by 6-months follow-up | -5.80 | 79.6 | .94 |
| Placebo + F-BA by 12-months follow-up | 1.17 | 78.0 | .99 |
| Supplements by 12-months follow-up | -117.0 | 79.5 | .142 |
| Supplements + F-BA by 12-months follow-up | -77.6 | 79.9 | .33 |
| Analyses are adjusted for age, gender, site, education, body mass index, smoking, alcohol use, physical activity, diabetes mellitus, heart disease, and other chronic somatic disorders. n-3: omega-3. n-6: omega-6. PUFA: polyunsaturated fatty acids. DHA: docosahexaenoic acid. EPA: eicosapentaenoic acid. F-BA: food related behavioral activation. | | | |
